# Metabolomics: insights into plant‐based diets

**DOI:** 10.15252/emmm.202013568

**Published:** 2021-02-01

**Authors:** Annamarie E Allen, Jason W Locasale

**Affiliations:** ^1^ Department of Pharmacology and Cancer Biology Duke University Durham NC USA

**Keywords:** Metabolism

## Abstract

Plant‐based diets exclude or substantially limit the consumption of meat and animal products and are of growing interest to many due to their sustainability and health benefits (Eshel *et al*, 2016). Veganism is an extreme type of plant‐based diet which excludes the consumption of all animal‐derived foods such as meat, eggs, and dairy, as well as foods containing animal‐derived ingredients. In adults, for example, certain observational studies have suggested lower body mass index, total cholesterol, LDL‐cholesterol, decreased incidence and mortality from ischemic heart disease, and decreased incidence of cancer in vegans and vegetarians versus omnivores (Dinu *et al*, 2017). The mechanistic basis for these observations and their generality are unclear.

## Graphical abstract

Allen A. and Locasale J. discuss the the metabolic and nutritional consequences of vegan diets in children, as reported by A. Suomalainen and colleagues, in this issue of *EMBO Mol Med*.

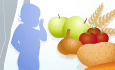

The direct action of metabolism, in addition to systemic effects such as hormonal regulation and inflammation, provides a link from diet to health. Many aspects of whole‐body metabolism can be inferred non‐invasively in humans by analyzing the metabolite composition of plasma, which is easily accessible and offers a whole‐body readout of many aspects of physiology. Due to differences in protein composition in these diets, amino acid intake and plasma levels may account for one of the main differences between vegan and vegetarian diets (Schmidt *et al*, 2015). Vegan diets are especially low in methionine which is highest in red meat, eggs, and dairy, and methionine restriction independently increases lifespan and causes some of the same health benefits as vegan diets such as cancer protection (Sanderson *et al*, 2019). Vegan diets have also been shown to change gut microbiome composition, and this may contribute to the plasma metabolome of vegans more so than omnivores (Wu *et al*, 2016).

While these and other studies have been conducted in adults, children have different nutritional requirements than adults, and the effects of vegan diets on children and associated recommendations are unclear. One study found vegan children tended to be smaller than non‐vegan children, but within normal ranges, and had deficits in calorie, calcium, and vitamin D intake (Sanders, 1988). The American Dietetic Association considers vegan diets safe for all age groups including children provided that they are well‐planned, whereas the German Nutrition Society does not recommend vegan diets for children. Guidelines for a vegan diet in children include consuming large amounts and a wide variety of plant foods, choosing vegetable fats selectively, consuming adequate amounts of calcium, zinc and iron, and supplementing vitamin D, B12, and the polyunsaturated fatty acid docosahexaenoic acid (DHA) (Baroni *et al*, 2018).

Besides assessing the ability of vegan diets to support normal growth and development, understanding the metabolic and physiologic effects of certain diets in children could offer a starting point to develop new strategies for dietary management of certain childhood disorders with limited pharmacologic treatment options. Areas of interest include inborn errors of metabolism, mitochondrial disease, and autism spectrum disorders (van Karnebeek & Stockler, 2012; Ahola *et al*, 2016; Vuong & Hsiao, 2017).

In this issue of *EMBO Molecular Medicine*, Hovenin *et al* (2021) begin to explore these open questions by analyzing dietary records and the serum metabolome of vegan, vegetarian, and omnivore children living in Finland and particularly focus on the differences between vegans and omnivores. They found that vegan children had a distinct metabolic profile which included differences in bile acid biosynthesis, circulating fatty acid levels including lower DHA, considerably lower levels of cholesterol and high‐ and low‐density lipoproteins, alterations in circulating amino acids, and lower levels of vitamins A and D that suggested possible rationale for additional supplementation (Fig 1).

**Figure 1:**
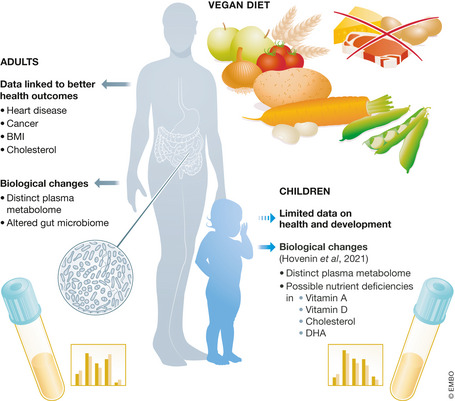
Vegan diets are defined by their exclusion of animal‐derived foods including meat, eggs, and dairy In adults, vegan diets have been linked to improved health outcomes and adults consuming a vegan diet show biological changes including distinct alterations in their plasma metabolome and gut microbiome. Less is known about how vegan diets affect children, and to this end, Hovenin *et al* (2021) explore the metabolic and nutritional effects of vegan diets in children.

Vegan children had lower protein intake calculated as a percentage of daily energy intake (13.5% compared to 16.4% in omnivores) and showed lower levels of circulating leucine/isoleucine, phenylalanine, valine/betaine, and aspartate and higher levels of alanine, arginine, and glycine. These alterations are generally in line with amino acid alterations seen in adult vegans, with the notable exception of unchanged serum methionine in vegan children. It remains to be seen whether consuming differing protein sources versus a lower percentage of energy consumption from protein contributed to the overall pattern of circulating amino acid levels in vegan children. It is also unclear whether the lower levels of several essential amino acids could constitute a deficiency or a health benefit, as to our knowledge there are no guidelines for healthy blood levels of individual amino acids and their effects on metabolism are still in its early stages. Perhaps relevant is the low levels of total cholesterol and high‐and low‐density lipoproteins seen in vegan children along with the indicators that there is not compensatory cholesterol biosynthesis. Blood cholesterol also does not have clearly defined deficiency levels, and as Hovenin *et al* (2021) note, cholesterol is required for cell membrane synthesis and steroid hormone synthesis among its other roles and may be required at higher levels during development.

Pathway analysis on the untargeted metabolomics data showed bile acid biosynthesis was the largest pathway alteration in vegan children. Pathway alterations included higher levels of unconjugated primary bile acids and a lower taurine to glycine conjugation ratio of bile acids in vegans than in omnivores, and however, authors note that it is unknown how these changes are likely to affect the roles of bile acids in digestion, absorption, endocrine, and gut microbiome–brain interactions.

In conclusion, Hovenin *et al* (2021) use metabolomics to study the metabolic and nutritional consequences of vegan diets in children and find similarities and differences to what has been previously observed in adults. This leads to many open questions as to how these types of diets might be used to affect specific aspects of metabolism and downstream health consequences such as development. Given the metabolic heterogeneity observed across the population, it’s unlikely that a vegan diet would be beneficial all in all settings. Nevertheless, certain children exhibited specific metabolic profiles that suggest that they could be specifically affected by the vegan diet. More work is needed to know whether such observations have molecular consequences and their generalizability.

## References

[emmm202013568-bib-0001] Ahola S , Auranen M , Isohanni P , Niemisalo S , Urho N , Buzkova J , Velagapudi V , Lundbom N , Hakkarainen A , Muurinen T *et al* (2016) Modified Atkins diet induces subacute selective ragged‐red‐fiber lysis in mitochondrial myopathy patients. EMBO Mol Med 8: 1234–1247 27647878 10.15252/emmm.201606592 PMC5090657

[emmm202013568-bib-0002] Baroni L , Goggi S , Battaglino R , Berveglieri M , Fasan I , Filippin D , Griffith P , Rizzo G , Tomasini C , Tosatti MA *et al* (2018) Vegan nutrition for mothers and children: practical tools for healthcare providers. Nutrients 11: 5 10.3390/nu11010005 PMC6356233 30577451

[emmm202013568-bib-0003] Dinu M , Abbate R , Gensini GF , Casini A , Sofi F (2017) Vegetarian, vegan diets and multiple health outcomes: a systematic review with meta‐analysis of observational studies. Crit Rev Food Sci Nutr 57: 3640–3649 26853923 10.1080/10408398.2016.1138447

[emmm202013568-bib-0004] Eshel G , Shepon A , Noor E , Milo R (2016) Environmentally optimal, nutritionally aware beef replacement plant‐based diets. Environ Sci Technol 50: 8164–8168 27387141 10.1021/acs.est.6b01006

[emmm202013568-bib-0005] Hovenin T , Korkalo L , Freese R , Skaffari E , Isohanni P , Niemi M , Nevalainen J , Gylling H , Zamboni N , Erkkola M *et al* (2021) Vegan diet in young children remodels metabolism and challenges the statuses of essential nutrients. EMBO Mol Med 13: e13492 10.15252/emmm.202013492 PMC7863396 33471422

[emmm202013568-bib-0006] van Karnebeek CD , Stockler S (2012) Treatable inborn errors of metabolism causing intellectual disability: a systematic literature review. Mol Genet Metab 105: 368–381 22212131 10.1016/j.ymgme.2011.11.191

[emmm202013568-bib-0007] Sanders TA (1988) Growth and development of British vegan children. Am J Clin Nutr 48: 822–825 3414589 10.1093/ajcn/48.3.822

[emmm202013568-bib-0008] Sanderson SM , Gao X , Dai Z , Locasale JW (2019) Methionine metabolism in health and cancer: a nexus of diet and precision medicine. Nat Rev Cancer 19: 625–637 31515518 10.1038/s41568-019-0187-8

[emmm202013568-bib-0009] Schmidt JA , Rinaldi S , Ferrari P , Carayol M , Achaintre D , Scalbert A , Cross AJ , Gunter MJ , Fensom GK , Appleby PN *et al* (2015) Metabolic profiles of male meat eaters, fish eaters, vegetarians, and vegans from the EPIC‐Oxford cohort. Am J Clin Nutr 102: 1518–1526 26511225 10.3945/ajcn.115.111989 PMC4658459

[emmm202013568-bib-0010] Vuong HE , Hsiao EY (2017) Emerging roles for the gut microbiome in autism spectrum disorder. Biol Psychiatry 81: 411–423 27773355 10.1016/j.biopsych.2016.08.024 PMC5285286

[emmm202013568-bib-0011] Wu GD , Compher C , Chen EZ , Smith SA , Shah RD , Bittinger K , Chehoud C , Albenberg LG , Nessel L , Gilroy E *et al* (2016) Comparative metabolomics in vegans and omnivores reveal constraints on diet‐dependent gut microbiota metabolite production. Gut 65: 63–72 25431456 10.1136/gutjnl-2014-308209 PMC4583329

